# Biocompatible Nanocarriers for Enhanced Cancer Photodynamic Therapy Applications

**DOI:** 10.3390/pharmaceutics13111933

**Published:** 2021-11-15

**Authors:** Sathish Sundar Dhilip Kumar, Heidi Abrahamse

**Affiliations:** Laser Research Centre, Faculty of Health Sciences, University of Johannesburg, Johannesburg 2028, South Africa; habrahamse@uj.ac.za

**Keywords:** photodynamic therapy, biocompatible, cancer, nanocarriers, photosensitizers

## Abstract

In recent years, the role of nanotechnology in drug delivery has become increasingly important, and this field of research holds many potential benefits for cancer treatment, particularly, in achieving cancer cell targeting and reducing the side effects of anticancer drugs. Biocompatible and biodegradable properties have been essential for using a novel material as a carrier molecule in drug delivery applications. Biocompatible nanocarriers are easy to synthesize, and their surface chemistry often enables them to load different types of photosensitizers (PS) to use targeted photodynamic therapy (PDT) for cancer treatment. This review article explores recent studies on the use of different biocompatible nanocarriers, their potential applications in PDT, including PS-loaded biocompatible nanocarriers, and the effective targeting therapy of PS-loaded biocompatible nanocarriers in PDT for cancer treatment. Furthermore, the review briefly recaps the global clinical trials of PDT and its applications in cancer treatment.

## 1. Introduction

Cancer is caused by the uncontrollable and abnormal proliferation of cells in the body, and is the leading cause of death worldwide [1]. Radiation and chemical carcinogens have been identified as significant causes of cancer that damage DNA and induce mutations in the cells, and different types of cancers have been identified [2]. Most types of cancer are highly resistant to conventional chemotherapy and radiotherapy, which is known for its elevated multidrug resistance and low rate of patient survival [3]. According to the World Health Organization, it is estimated that there were nearly 10 million cancer deaths in 2020, and the most common causes of cancer death in 2020 were lung, colon and rectum, liver, stomach, and breast cancer [4]. Identifying novel therapy for the successful treatment of cancer is a significant problem across the world. Chemotherapy is widely used as a conventional cancer therapy: the primary function of this therapy is to destroy cancer cells, but it often kills both cancer and normal cells [5]. Chemotherapy is often associated with severe side effects to the patient’s health, and some of the normal cells (blood-forming cells in the bone marrow, hair follicles, and cells in the mouth, digestive tract, and reproductive system) are the most likely to be damaged [6]. Hence, developing a targeted therapy approach is the best way to destroy cancer cells instead of normal cells.

In the past few decades, nanotechnology has received substantial attention amongst scientists to understand tumor-targeted nanoparticle-mediated drug delivery for cancer therapy [7]. Biocompatible nanocarriers can be successfully used as a carrier molecule for various biomedical applications, including drug delivery [8], phototherapy [9] biosensing [10], bioimaging [11], diagnosis [12], cancer therapy [13], and hyperthermia [14]. The development and employment of biocompatible nanocarriers provide various functional applications in the treatment of cancer, such as a controlled delivery of drugs, being non-immunogenic, a long shelf life, more excellent stability, efficient drug loading, functionalization of targeting ligands, and intracellular drug release into targeted cells. Biocompatible nanocarriers exhibit good cytocompatibility and hemocompatibility, are friendly to physiological conditions, they should be non-toxic when entering the body, and they contact tissues and cells directly [15,16,17]. The toxic nanoparticles are often called non-biocompatible materials. Various factors are associated with the toxic effects of nanoparticles, such as nanoparticle concentration, interaction time with living matter, stability in biological fluids, and accumulation capacity in tissues and organs. The complete understanding of the factors mentioned above and their positive outcomes help develop a safe and biocompatible nanoparticle for drug delivery applications [18]. Biocompatible nanocarriers-based photosensitizer (PS) delivery has become a promising platform for PDT, and it offers many benefits owing to its unique physicochemical properties [19].

Furthermore, nanoparticle-based delivery enables passive drug targeting by utilizing the enhanced permeability and retention effect (EPR effect) in cancerous tissues [20]. The potential role of functionalized nanomaterials in cancer photodynamic therapy (PDT) and their effective targeting efficiency have been extensively studied [21,22,23]. Photodynamic therapy is an emerging, minimally invasive therapeutic strategy for the management of a variety of cancers. Photodynamic therapy involves three principal components: PS; visible light; and oxygen—the activated PS helps the formation of ROS, and initiates the cytotoxic effects in the target cells or tissues [24]. Photodynamic therapy has been investigated for more than 25 years as an unconventional treatment for cancer [25]. The first PS introduced by Dougherty et al. (1975) was a water-soluble mixture of porphyrins named “haematoporphyrin derivatives” (HpD), and the modern term, “photofrin”. Several light-absorbing and light-activating compounds are used as PS in PDT, and it has shown significant outcomes in cancer treatment [26]. The photochemical and photophysical properties of PS-loaded biocompatible nanocarriers play a vital role in the cancer cell death mechanism. Briefly, the PS is activated and excited at a specific wavelength to induce cell death through the production of reactive oxygen species (ROS) [27,28] 

In this review article, we explore the use of biocompatible nanocarriers-based PS delivered in PDT for cancer treatment. Thus, this review article is divided into six different sections. Section 1 briefly explains the introduction to the study. Section 2 studied the use of different biocompatible nanocarriers and their potential applications in PDT. In Section 3, PS-loaded with biocompatible nanocarriers in PDT for cancer are discussed in detail. Section 4 describes the effective targeting approach of PS-loaded biocompatible nanocarriers in PDT for cancer, and some of the global clinical trials of PDT in cancer treatment are given in Section 5. In Section 6, future perspectives of this work are briefly discussed.

## 2. The Potential Application of Biocompatible Nanocarriers in PDT

**Figure 1:**
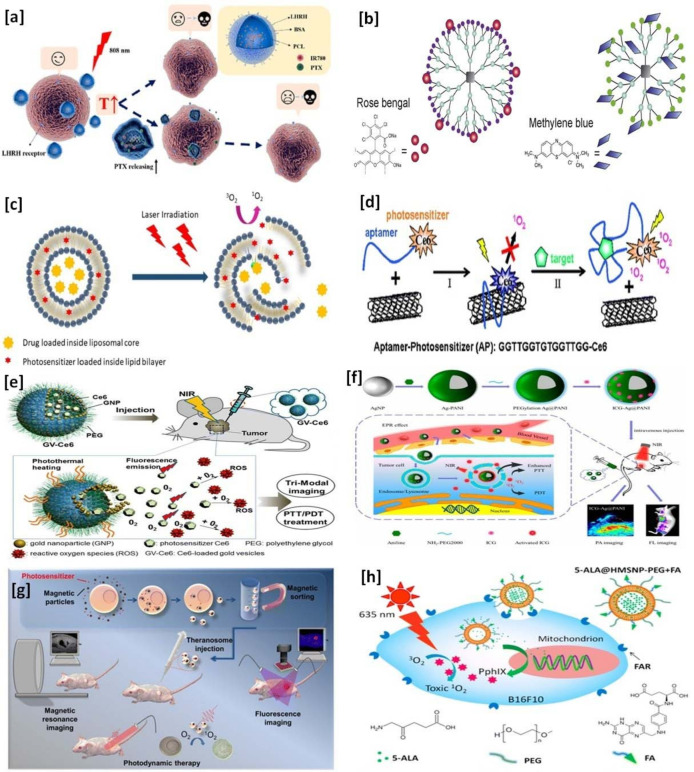
Schematic illustration of photosensitizers-loaded nanocarriers in PDT for the treatment of cancer: (**a**) polymeric nanoparticles—reprinted with permission from reference [84], Copyright 2020, American Chemical Society; (**b**) dendrimers—reprinted with permission from reference [85], Copyright 2015, Elsevier; (**c**) liposomes—reprinted with permission from reference [86], Copyright 2019, Elsevier; (**d**) carbon-based nanoparticles—reprinted with permission from reference [87], Copyright 2008, American Chemical Society; (**e**) gold nanoparticles—reprinted with permission from reference [70], Copyright 2013, American Chemical Society; (**f**) silver nanoparticles–reprinted with permission from reference [88], Copyright 2016, American Chemical Society; (**g**) magnetic nanoparticles—reprinted with permission from reference [89], Copyright 2013, American Chemical Society; and (**h**) mesoporous silica nanoparticles—reprinted with permission from reference [90], Copyright 2015, American Chemical Society.

The functional role of biocompatible nanocarriers and their applications in PDT are widely reported for cancer treatment. There are different types of biocompatible nanocarriers studied in cancer PDT—some are polymer-based nanocarriers [29], dendrimers [30], liposomes [31], carbon-based nanoparticles [32], gold nanoparticles [33], silver nanoparticles [34], magnetic nanoparticles [35], and mesoporous silica nanoparticles [36]. Schematic illustrations of PS-loaded nanocarriers in PDT for the treatment of cancer are given in Figure 1.

### 2.1. Polymer-Based Nanocarriers

The polymer-based nanocarriers are more often considered as carrier molecules for various drug delivery applications, including PDT. Polymer-based nanocarriers can alter their surface chemistry to functionalize the specific targeting moieties (ligands or antibodies), which helps to facilitate site-specific or targeted drug delivery to the site of action [37,38]. Li et al. (2021) recently reported the use of biocompatible nanocomplex for cancer therapy—the synthesized 5,10,15,20-tetrakis (4-aminophenyl) porphyrin (TAPP) and Fe3^+^-loaded PEGylated polygalacturonic acid nanocomplex increased the ^1^O_2_ upon 760 nm laser irradiation, and exhibited cytotoxic effects in B16 melanoma cells [39]. Chlorin e6-loaded poly (dopamine) nanospheres showed enhanced PDT/PTT effects in tumor cells upon 670 nm and 808 nm laser irradiation. In 670 nm laser irradiation, the poly (dopamine) conjugated chlorin e6 exhibited higher PDT efficacy, cellular update, and ROS production in tumor cells compared to free chlorin e6. It was also observed that the synthesized nanospheres showed an excellent PTT effect upon 808 nm laser irradiation. The combination of 670 and 808 nm irradiation confirmed the low dark toxicity with excellent phototoxicity effects of the nanospheres in both in vitro and in vivo studies [40]. Zinc Phthalocyanine(ZnPc)-loaded carboxymethyl chitosan/ionic liquid imidazolium-based nanoparticles in an in vitro release study confirmed the sustained release of ZnPc. The synthesized nanoparticles showed great ability to generate singlet oxygen, and it is appropriate to facilitate the high PDT activity to diseased cells [41]. Light-responsive polymers have attracted considerable attention in PDT, and are designed to load and release the encapsulated drugs inside the cells. An external light triggers the light-responsive polymers to release the drugs in both in vitro and in vivo systems by inducing the local heating, leading to thermal degradation of the matrix [42]. Different light parameters, such as wavelength, power, and pulse length, play an essential role in the triggering process, and the optimized parameters may induce disruption on the chemical structures (breaks covalent bonds) of polymers [43]. Schoppa et al. (2021) recently synthesized the light-responsive polymeric nanoparticles using the emulsion diffusion method with an average hydrodynamic diameter of 200 nm, and the negative zeta potential shows excellent colloidal stability. The light-responsive property and nanoparticle degradation study showed that the reduction of the molar mass of the polymer was confirmed by size exclusion chromatography upon light irradiation. The light-depending drug release property was evaluated using the PS 5,10,15,20-tetrakis (m-hydroxyphenyl) chlorine (mTHPC), and it confirmed the improved intracellular uptake of mTHPC in the colon cancer cell line (HT-29 cells) [44].

### 2.2. Dendrimers

Dendrimers are three-dimensional, branched macromolecules with a low polydispersity index, and they have shown numerous advantages as carrier molecules in drug delivery due to their physicochemical and biological properties. The three-dimensional structure consists of an initial core with repeated branching and terminal groups connected to the units of repeated branching, and this dendron-like structure ensures good stability, easy surface modifications, and biocompatibility for different drug delivery applications [45,46,47]. The structural morphology of dendrimers offered more inner space to load drug molecules/PS, and it facilitated passive targeting to the cells [48]. Dendrimers can be successfully used as a stabilizer for metal-based nanoparticles, and it shows numerous advantages in PTT by enhancing the high photothermal conversion efficiency of the nanoparticle upon irradiation [49]. Rose bengal is successfully loaded on cationic phosphorous dendrimer complex, and it showed enhanced production of singlet oxygen molecules in the aqueous medium than free rose bengal [50]. Zhou et al. (2018) synthesized water-soluble monodisperse dendritic carriers by incorporating 5-Aminolevulinic acid (ALA) and iron-chelating agents. The lowest concentration of 2 µM showed efficient phototoxicity in human epithelial carcinoma cells (KB cells), and the synergistic effects of iron chelating agents and ALA efficiently increased the intracellular accumulation of protoporphyrin IX [51]. Kojima et al. (2007) synthesized PS-loaded poly (ethylene glycol) (PEG), attached poly (amidoamine), PAMAM dendrimer, and PEG attached poly (propylene imine) (PPI) dendrimers for PDT applications. The inner hydrophobicity of PS-loaded PEG-PPI dendrimers showed enhanced stability compared to PEG-PAMAM dendrimers. An in vitro study revealed that the protoporphyrin IX(PpIX)-loaded PEG-PPI showed a better cytotoxic effect than free PpIX upon light irradiation [52].

### 2.3. Liposomes

Liposomes are biocompatible and biodegradable colloidal vesicles, and this characteristic feature makes this material more flexible to use as a carrier molecule in drug delivery applications [53]. Liposomal formulations can accommodate PS with variable physicochemical properties, substantially protect PS safety, and improve their PDT efficacy [54]. These conventional liposomes have limitations, especially the plasma half-life and difficulty loading and delivering the drug to the targeted area. Surface-modified liposomes have often overcome those limitations, and they ensure the safety and delivery of loaded drugs to the targeted tumor site [31]. An in vivo Meth-A sarcoma-bearing mice study showed that the pegylated liposomes enhanced the accumulation of benzoporphyrin-based PS in tumor tissues. The targeting delivery of surface-modified pegylated liposomes strongly suppressed the tumor growth, and then, the pegylated liposomes upon laser irradiation. The surface-modified pegylated liposomes also show a four-fold higher delivery of benzoporphyrin-based PS than the non-modified liposomes [55] Yang et al. (2019) synthesized aggregation-induced emission PS entrapped liposomes for PDT applications. The study results confirmed that the liposomes act as the most suitable carrier for PS delivery, and greatly improved the tumor-targeting behavior of liposomes [56]. The physicochemical characteristics of liposomes, such as thermodynamic phase and self-assembling properties, allow them to load and have a site-specific delivery of both hydrophilic and lipophilic drugs [57]. Feuser et al. (2021) successfully achieved the co-encapsulation of sodium diethyldithiocarbamate (DETC) and zinc phthalocyanine (ZnPc) in liposomes above 85% through the reverse-phase evaporation method. The synthesized liposomes showed a 308 nm diameter mean size with a highly stable zeta potential of −36 mV. The cytotoxic effects of co-encapsulation were studied against mouse embryo fibroblast (NIH3T3) cells, and it showed decreased cellular effects compared to free DETC+ZnPc. Enhanced phototoxic effects of the co-encapsulated liposomes were observed against human breast cancer cells (MDA-MB231) [58]. Lee et al. (2019) confirmed that the chitosan-coated liposomes protected the stability and skin permeation of indocyanine green better than uncoated liposomes. An in vitro B16-F10 melanoma cells study demonstrated that the chitosan-coated liposomes enhanced indocyanine’s cellular uptake and photocytotoxicity effects upon 775 nm laser irradiation at a power of 250 mW for 2.5 min [59]. The liposomal formulation plays a significant role, and it helps to use a lesser concentration of PS for PDT applications. Miretti et al. (2020) synthesized dipalmitoylphosphatidylcholine cholesterol liposomes, and successfully loaded different PS (Zinc Phthalocyanines and its derivatives, Zn(II)tetraminephthalocyanine), and the PDT efficiency of PS-loaded liposomes were studied using a glioblastoma cell line (T98G cells). The results showed that the liposomal formulations with 0.05 µM concentration of PS achieved similar cellular effects to that of the 0.5 µM concentration [60].

### 2.4. Carbon-Based Nanoparticles

The promising chemodynamic therapy (CDT), PDT, photothermal therapy (PTT) effects of Fe-N codoped carbon nanoparticles were investigated in both in vitro and in vivo models. The photothermal effects of synthesized nanoparticles were studied by irradiating with an 808 nm (1.5 W/cm^2^) laser, and it showed excellent photothermal conversion efficiency (around 29.15%), enhanced OH production, and H_2_O_2_ enhanced ROS generation in in vitro. Further, the synergistic CDT/PDT/PTT effects of nanoparticles were confirmed using 4T1 tumor-bearing Balb/c mice as an in vivo model. A 21-day mice study demonstrated that the synthesized nanoparticles can control the tumor growth, and it was observed that the tumor weight was reduced approximately 53 times smaller than the PBS (-) group. The treatment approaches also ensure the low biotoxicity of nanoparticles in in vivo, and there were no noticeable weight changes and histopathological damages in all the studied groups [61]. Zheng et al. (2016) synthesized protoporphyrin-loaded and Arg-Gly-Asp (RGD) targeting motif coated carbon nitride-based multifunctional nanocomposite for enhanced PDT. It was confirmed that the synthesized nanocomposites accumulated in the tumor tissues through active RGD targeting and the passive EPR effect. Then, the nanocomposite started splitting water to generate O_2,_ and the PS generated singlet oxygen (^1^O_2_) from the produced O_2_ upon 630 nm laser irradiation. An in vivo biodistribution study revealed that the targeting specificity of synthesized nanocomposite and the nanocomposite accumulation was explicitly observed in the tumor region. It was confirmed through the confocal laser scanning microscope study, by observing a strong fluorescence signal in the tumor region, and a similar signal was absent in metabolic organs, such as the lung, liver, and kidney [62]. Chan et al. (2016) synthesized noncytotoxic graphitic carbon nitride quantum dots (g-C_3_N_4_ QDs) using a solid-phase hydrothermal method as a potential PS for cancer treatment. The synthesized g-C_3_N_4_ QDs exhibit limited tissue permeability upon ultraviolet light radiation, while also having excellent cytotoxic properties. The modified form of g-C_3_N_4_ QDs nanocomposite using NaYF4:Yb/Tm and poly(L-lysine) produces ROS by entering into the cells, is responsible for the damage of mitochondrial function, and is associated with the induction of apoptosis in an oral epidermoid carcinoma cell line (OEC-M1) [63].

### 2.5. Gold Nanoparticles

The unique optical property and the biocompatibility of gold nanoparticles received considerable attention for cancer PDT treatments [64]. Gold nanoparticles require either covalent or non-covalent attachment approaches to deliver the therapeutic molecules in drug delivery, targeting, and imaging applications [65]. Photosensitizer-loaded gold nanoparticles can be successfully used to deliver to the tumor site via active or passive accumulation [66]. Cheng et al. (2011) performed an in vivo animal experiment to study PEGylated gold nanoparticles’ drug release mechanism and pharmacokinetics. The intravenous administration of gold nanoparticles showed an efficient PDT drug release and penetration into the tumor site without producing any adverse effects of the synthesized molecules. An in vivo fluorescence imaging study confirmed the fast drug excretion from the body by renal clearance and the hepatobiliary system [67]. Dutta et al. (2019) synthesized cationic PS methylene blue(MB)-loaded gold nanocluster embedded mucin nanoparticles by the facile green synthesis method for an imaging-guided PDT application. The synthesized nanoparticles showed an average size of 139 ± 47 nm, and the size of the gold cluster revealed an average size of 1.9 ± 0.34 nm inside the mucin nanoparticles. The synthesized nanoparticles show a sustained release pattern, with a higher MB release in acidic pH 4.5, and a lesser release in pH 7.4 (24 h), and it directly indicates the beneficial release behavior of synthesized nanoparticles in the tumor microenvironment. The cell viability study revealed the biocompatibility nature of nanoparticles against HeLa cells (human cervical carcinoma) and HEK 293T (human embryonic kidney) cells. The luminescent property of synthesized nanoparticles aids in understanding the bioimaging of PS inside the cells, and it can be successfully used for bioimaging guided cancer PDT [68]. The combined cancer PDT and PTT effects of covalently immobilized porphyrin-loaded crosslinked polyphosphazene nanospheres based on gold nanoparticles were studied by Wei et al. (2018). The synthesized nanoparticles showed low cytotoxicity against HeLa cells in the dark, and showed significant cytotoxicity upon laser irradiation with 808 nm (PTT) and 630 nm (PDT) [69]. Chlorin e6(Ce6)-loaded gold vesicles showed strong absorption in the range of 650–800 nm, and it ensures the enhanced cellular update and localization of Ce6 in human breast cancer cells (MDA-MB-435). The in vitro cell viability of laser irradiated gold vesicles, Ce6, and synthesized Ce6-loaded gold vesicles were analyzed by MTT assay. The results indicate that the cytotoxic effects of Ce6-loaded gold vesicles show a higher percentage (45–70%) than cells treated with gold vesicles (5%) and Ce6 (10%). An in vivo study performed using tumor-bearing mice shows the higher therapeutic effect of Ce6-loaded gold vesicles than gold vesicles and Ce6. An in vitro and in vivo study suggested that the synthesized Ce6-loaded gold vesicles may be suitable for image-guided synergistic PTT/PDT cancer treatment [70].

### 2.6. Silver Nanoparticles

Silver nanoparticles are broadly studied for their various biomedical applications, including drug delivery, PDT, antimicrobial activity, and cellular imaging. Silver nanoparticles were irradiated using a 635 nm diode laser, and it showed decreased cell viability, proliferation, and induced apoptosis (programmed cell death) in breast cancer cells (MCF-7) and lung cancer cells (A549). Although cell death was caused in both cell lines, the AgNPs exhibited enhanced cytotoxic effects in the MCF-7 compared to A549 cells [71]. This indicates that different types of cancer cells respond differently to the same NPs, and thus, studies on various cancer cell types need to be examined. Mahajan et al. (2019) synthesized porphyrin-loaded mercaptosuccinic acid capped silver nanoparticles for PDT and cell imaging applications. The average size distribution of synthesized nanoparticles shows 110 nm with excellent zeta potential (−31.1 mV), and the scanning electron microscopy study shows the uneven cube synthesized nanoparticles were studied against A375 malignant melanoma cancer cells. It shows non-toxic behavior up to the concentration of 5 µM, with excellent fluorescence imaging properties [72]. Natesan et al. (2017) synthesized hypocrellin B and nanosilver-loaded poly lactide-co-glycolide-based nanoparticles by a nanoprecipitation method for enhanced production of singlet oxygen (^1^O_2_), and it showed the time-dependent phototoxicity against A549 (human adeno lung carcinoma) cells upon irradiation [73]. Curcumin and silver nanoparticles-loaded chitosan and chondroitin sulfate-based hydrogels were synthesized by Fabiano de Freitas et al. (2020), and it was further modified with ionic liquids to enhance the solubility properties of chitosan. The cytotoxic and phototoxic behaviors of synthesized nanoparticles were analyzed using fibroblast cell lines (CCL1.3). The cytotoxic results exhibited >80% cell viability for the studied highest concentrations (1000 µg mL^−1^), and the phototoxicity assay showed cell death at ~60% at the highest concentrations. There was no cell death in the absence of the loaded molecules (curcumin and silver nanoparticles) [74].

### 2.7. Magnetic Nanoparticles

Magnetic nanoparticle-based carriers have been extensively studied for their applications in biomedicine, such as drug delivery, drug targeting, cell isolation and sorting, magnetic resonance imaging, and cancer PDT [75]. Iron oxide-based materials are considered an excellent choice to use as magnetic nanoparticles due to their already proven biocompatibility and superparamagnetic behavior [76]. Protoporphyrin IX PS-loaded multifunctional magnetic nanoparticles were synthesized using triphenylphosphine (TPP)-grafted dextran to enhance the magnetic imaging-guided PDT effect in tumor cells. The average particle size distribution of synthesized nanoparticles was found in the range of 76.91 ± 5.33 nm, with corresponding polydispersity indices of 0.223, and negatively charged zeta potential (−5.73 ± 0.65 mV). The cellular uptake and photoinduced internalization of synthesized nanoparticles were studied in 4T1 cells (mouse breast tumor cells), and it confirmed the enhanced effect compared to control, and, further, the mitochondrial damage was confirmed using a fluorescence microscopy study. An in vivo study, performed using 4T1 tumor-bearing Balb/c mice, indicates the antitumor effect upon laser irradiation [77]. Cinteza et al. (2006) synthesized the 2-[1-hexyloxyethyl]-2-devinyl pyropheophorbide-a (HPPH) PS-loaded diacyllipid micelle-based magnetic Fe_3_O_4_ nanoparticles. The synthesized nanoparticle shows excellent stability and physicochemical properties for magnetic guided PDT drug delivery and light-activated PDT. It also exhibits an enhanced in vitro cellular uptake and phototoxicity against HeLa cells [78]. Di Corato et al. (2015) synthesized dually-loaded hybrid liposomes for PDT and magnetic hyperthermia applications. They successfully loaded iron oxide nanoparticles and PS (m-Tetrahydroxyphenylchlorin (m-THPC)) within the aqueous core and lipid bilayer, respectively, by a one-pot synthesis method. An in vitro antitumoral efficacy of synthesized nanoparticles was performed using human adenocarcinoma SKOV-3 cells, and it created tumor cell death when each treatment was performed alone, and the combined treatment revealed the destruction of cells in vitro. Similarly, an in vivo study was conducted using six-week-old female NMRI nude mice, and the synthesized nanoparticles were capable of inhibiting tumor growth when each treatment was performed alone, and the combined treatment revealed the complete eradication of the tumor [79]. Superstable magnetic nanoparticles were used in conjugation with indocyanine green (IR820), a near-infrared organic dye used as a PS for PDT applications. The synthesized PS-loaded nanoparticles exhibit excellent stability in water, and an enhanced PDT efficiency was observed compared to free IR820, and it showed improved cellular uptake and fluorescence intensity of the cells [80].

### 2.8. Mesoporous Silica Nanoparticles

Biocompatible and biodegradable mesoporous silica-based nanoparticles have been extensively studied in drug delivery applications owing to their physicochemical properties, such as tunable size, large surface area, biosafety, and surface modification flexibility. The tunable size and large surface area properties facilitate the load of one or more drug molecules, and the surface modification flexibility helps control the drug release behavior precisely [81]. Doxorubicin and rose-bengal-loaded hollow mesoporous silica nanoparticles (HMSNs) are successfully used in chemo-photodynamic combination therapy. The drug entrapment efficiency was 76.67% for doxorubicin, and 95.85% for rose bengal, and an in vitro drug release study indicated the pH-responsive drug release from HMSNs. The synthesized HMSNs show negligible drug release in pH 7.4 due to their tightly capped surface, and offer an improved and sustained drug release behavior in acidic pH (6.0 and 5.0). The synthesized nanoparticle shows excellent physicochemical properties, and the Fourier transform infrared spectroscopy study results confirmed the coating of hyaluronic acid on the surface of HMSNs. An in vitro cellular uptake study was performed using a confocal laser scanning microscopic technique to confirm the targeting ability of hyaluronic acid-coated HMSNs against murine mammary carcinoma (4T1 cells), which are considered CD44 receptor overexpressed cells, and hyaluronic acid has a high affinity on CD44 receptor [82]. Han et al. recently synthesized the ferrocene modified multifunctional mesoporous silica nanoparticles, and successfully loaded indocyanine green PS for synergistic CDT/PDT/PTT applications. An in vitro cellular study was performed using HeLa cells to determine the cell killing rate, and it was observed that 93% of cells were killed using the synergistic CDT/PDT/PTT approach. The loaded indocyanine green facilitates the generation of hyperpyrexia upon near-infrared light irradiation, and aims to kill the cancer cells using PTT. Ferrocene modification helps initiate an intracellular Fenton reaction, and kills the cells through CDT and generated singlet oxygen. Indocyanine green kills the cancer cells by PDT [83].

## 3. Biocompatible Nanocarriers for Photosensitizers in PDT

The design and exploration of various PS-loaded biocompatible nanocarriers in PDT have shown minimized toxicity in cancer therapy [91]. There are several PS compounds loaded with biocompatible nanocarriers studied in PDT and listed in Table 1.

## 4. Biocompatible Nano Carrier-Based Targeted Therapy in PDT

**Figure 2:**
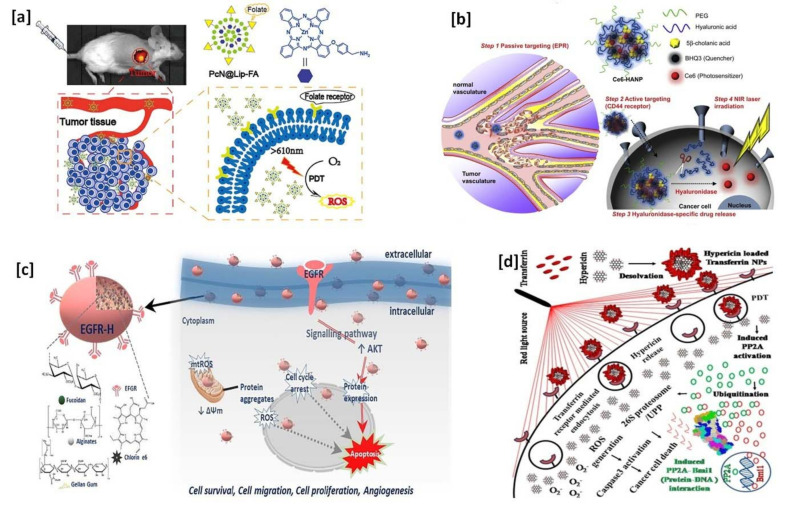
Schematic illustration of photosensitizers loaded cancer cell targeting efficiency of biocompatible nanocarriers in PDT: (**a**) folate receptor targeting—reprinted with permission from reference [115], Copyright 2020, Elsevier; (**b**) CD 44 receptor targeting—reprinted with permission from reference [116], Copyright 2012, Elsevier; (**c**) EGFR receptor targeting—reprinted with permission from reference [117], Copyright 2020, Elsevier; and (**d**) transferrin receptor targeting—reprinted with permission from reference [118], Copyright 2020, American Chemical Society.

Many types of cancerous cells overexpress some specific receptors on the cell surface—some of them are the folate receptor, CD 44 receptor, avβ3 integrin receptor, EGFR receptor, transferrin receptor, and epithelial cell adhesion molecules (EpCAM), also known as the CD326 receptor. The targeting ligand-modified biocompatible nano carrier-based approaches can target the site of action in PDT cancer therapy. Biocompatible nano carrier-based targeted therapy enhances the PDT effects by targeting such receptors using specific ligands. The detail of PS-loaded and targeting ligand modified nanocarriers and their great targeting potential in cancer treatments are discussed in Table 2. A schematic illustration of PS-loaded cancer cell targeting efficiency of biocompatible nanocarriers in PDT are given in Figure 2.

## 5. Clinical Application of PDT in Cancer Treatment

Photodynamic therapy-based cancer treatment shows several advantages, such as reduced toxicity and invasiveness, short treatment time, and being inexpensive compared to classical cancer therapies [119,120]. Biocompatible nanocarriers and several PSs are well studied in global clinical trials, and it has shown positive effects in cancer PDT. Some of the significant global clinical trial statuses, and details of the PS and PDT parameters, are given in Table 3.

## 6. Future Perspectives and Conclusions

In this review article, we discussed the potential benefits of biocompatible nanocarriers and their enhanced cancer PDT applications. Different types of biocompatible nanocarriers are extensively studied in cancer PDT applications, and are well documented in the literature—some of them are polymer-based nanocarriers, dendrimers, liposomes, carbon-based nanoparticles, gold nanoparticles, silver nanoparticles, magnetic nanoparticles, and mesoporous silica nanoparticles. A comprehensive literature study revealed that the advancement of using biocompatible nanocarriers and their cancer PDT applications had shown numerous positive outcomes in both in vitro and in vivo studies. We strongly believe that the discussed biocompatible nanocarriers have shown numerous advantages for cancer PDT applications. Some of them include: targeted PS delivery to the site of action, ensuring the long-term stability of PS; enhanced loading efficiency; and the sustained release of PS. The successful preparation of biocompatible nanocarriers significantly avoids the limitations associated with chemo drugs and PS. We expect the use of photosensitizers loaded with biocompatible-based nanocarriers will be broader in the future, as well as more significant outcomes in clinical trials for cancer PDT applications.

## Figures and Tables

**Table 1:** Different types of photosensitizers-loaded biocompatible nanocarriers in PDT for cancer.

Biocompatible Nanocarriers	Preparation Method	Size (nm)	Photosensitizers	Outcomes
Albumin nanoparticles	Solvent diffusion method	100 to 200 nm	Hematoporphyrin	The synthesized hematoporphyrin-loaded albumin nanoparticle accumulation was increased in murine lung tumor cells compared to normal lungs cells [92].
Polymeric micelles (Pluronic P123 and F127 mixture)	Solvent evaporation method	12.5 to 16.6 nm	Photofrin II^®^	PDT irradiation on PS loaded polymeric micelles showed an increased cytotoxic effect in the human cancer cell model [93].
TmPyP-loaded PLGA nanoparticles	Evaporation method	Between 118 ± 5 and 133 ± 2 nm	5,10,15,20-tetrakis(1-methylpyridinium-4-yl)-porphyrin tetra-iodide (TMPyP)	The formulation showed positive outcomes in laser irradiation and skin permeability studies, and it can be successfully used for topical diseases, such as melanoma [94].
Chitosan nanoparticles	Ionic crosslinking method	254.3 ± 9.42 nm	ALA	The synthesized nanoparticle shows a spherical shape, good dispersion, and stability. The PDT effect of ALA-loaded nanoparticles was studied against WSU-HN6 and CAL-27 cells—the elevated mitochondrial ROS production was observed in both cells [95].
Thermoresponsive solid lipid nanoparticles	High-performance hot homogenization and ultrasonication method	from ~20 nm up to 700 nm	Temoporfin	Temoporfin-loaded solid lipid nanoparticle formulation was tested in 4T1 (murine mammary carcinoma) and MDA-MB-231 (human breast adenocarcinoma) cells. It showed faster accumulation in the cells, and induced increased phototoxicity against tumor cells [96].
Poly(d,l-lactide-co-glycolide) nanoparticles	Salting-out technique	Two types 167 and 370 nm in diameter	Verteporfin	The synthesized biocompatible polymeric nanoparticle was tested against EMT-6 mammary tumor cells, and the smaller size of the nanoparticle showed very good photocytotoxicity compared to large nanoparticles. Similarly, the small nanoparticles effectively controlled the tumor growth in an in vivo mice study [97].
Hyaluronic acid-based carbon nanotubes	π-π interactions	203 ± 6.6 nm	Chlorin e6	The synthesized single-walled carbon nanotubes confirmed the enhanced PDT effect of chlorin e6 against CACO-2 cells compared to free chlorin e6 [98].
Core-shell polymeric nanoparticles	Microemulsion polymerization method	~170 and 220 nm	HPPH	The synthesized nanoparticles help to prevent the fluorescence quenching in water. It helps to achieve fluorescence imaging-guided PDT [99].
Lipid polymer hybrid nanoparticles	Self-assembly	170 ± 20 nm	Zinc phthalocyanine	The synthesized lipid polymer hybrid nanoparticles improved the stability, cellular uptake, sustained release, and fluorescence properties of Zinc Phthalocyanine. The synthesized nanoparticle was tested both in vitro and in vivo. In vitro cytotoxic study shows increased cell death against MCF-7 cells, and an increased PDT antitumor effect in an in vivo study (Sprague Dawley rats) [100].
Pluronic-based nanocomposite	Thin-film hydration method.	121.8 nm	Methylene blue	The synthesized nanocomposite shows synergistic effects (PDT/PTT) against the human cervical cancer cell line (SiHa). Cell death occurred by following the cell apoptosis pathway, and it can effectively treat cancer via noninvasive phototherapy [101].
Multifunctional mesoporous silica nanoparticle	Sol-gel method	200 nm	Indocyanine green	The combined chemodynamic/PTT/PDT therapy shows that an increased inhibition rate of HeLa cells compared to the treatment given by chemodynamic therapy alone or dual PTT/PDT [83].
Rose bengal-loaded nanostructured poly-amidoamine dendrimers	Michael addition method followed by encapsulation	20 nm	Rose bengal	The controlled release property of Rose bengal-loaded dendrimer formulation was confirmed by the in vitro drug release study. The nanostructured formulation produced remarkable photocytotoxicity properties against DLA cells (Dalton’s Lymphoma Ascite) [102].
BODIPY with mPEG-based phototheranostic nanoparticle	Freeze-drying method	282 nm	BODIPY	The synthesized Mitomycin C-graphene BODIPY-mPEG nanoparticle possessed excellent properties for applying tumor tissue imaging-guided photo chemo synergistic therapy [103].
Pluronic^®^-based nanoparticles	Solid dispersion method	NA	Hypericin	The synthesized micelles showed high stability and selective internalization in MCF-7 cells. The accumulated micelles were observed in mitochondria and endoplasmic reticulum, and it showed effective phototoxic cell death [104].
Hypocrellin and nanosilver-loaded PLGA-TPGS copolymeric nanoparticles	Ring-opening and bulk polymerization method	89.59 to 566.8 nm	Hypocrellin	An enhanced phototoxic effect was observed in A549 cells (human adeno lung carcinoma) irradiated by 590 nm using a mercury vapor lamp [105].
Pectin-coated silver nanoparticles	Heated and stirring method	2.3 ± 0.7 nm and 9 ± 6 nm.	Riboflavin	The synthesized pectin-based nanoparticle increases the biocompatibility of silver nanoparticles, and the loaded riboflavin emission enhanced singlet oxygen production compared to the control. Cytotoxicity study shows the increased photodamage effect when nanoparticles and riboflavin are present in the sample [106].
Albumin-based nanoparticle	Self-assembly method	36 nm	Curcumin	Enhanced antitumor activity was observed in HeLa cells through PDT. The curcumin derivative-loaded nanoparticle induced cell cycle arrest and apoptosis in HeLa cells [107].

**Table 2:** Cancer targeting efficiency of biocompatible nanocarriers in PDT.

Name of the Nanocarriers/Targeting Ligand/Photosensitizers/Drug Used	Target	Cancer Treatment (In Vitro/In Vivo)	Outcomes	Reference
Folic acid-functionalized and Poly-lactic acid (PLA) coated, Indocyanine green loaded colloidal gold nanobipyramid	Folate receptor	Murine melanoma B16-F10 cell line (in vitro)	The synthesized nanocarriers targeted the overexpressed folate receptor on the membrane of B16-F10 cells, which also showed improved photothermal and photodynamic activity when irradiated with both 785 and 808 nm lasers.	[108]
Iridium (III) nano-PS self-assembled with hyaluronic acid (HA)	CD 44 receptor	Mouse metastatic breast cancer cells (4T1.2) (in vitro)	Nano-PS treated cells were irradiated by 532 nm light. The CD 44 receptor targeting efficiency of HA-coated nanoparticles showed excellent cellular uptake and mitochondria accumulation abilities, and it significantly improved the phototoxicity in 4T1.2 cells.	[109]
Protoporphyrin IX-loaded hyaluronic acid-based polymeric micelles	CD 44 receptor	CD44 overexpressing A549 cells (in vitro)	It is observed that the synthesized micelles showed an increased cellular uptake and enhanced phototoxic activity in CD44, overexpressing A549 cells in both 2D and 3D cultures.	[110]
Arg-Gly-Asp (RGD) peptide-functionalized chlorin e6 (Ce6) loaded PEGylated mesoporous silica nanoparticles	avβ3 integrin	The human glioma cell line of U87 MG cells (in vitro)	Confocal laser scanning microscopy study confirmed the cellular targeting efficiency and cellular internalization of RGD functionalized nanoparticles in U87 MG cells. 660 nm laser irradiation on RGD functionalized nanoparticles resulted in improved cellular toxicity than free Ce6.	[111]
Curcumin-loaded Poly (D, l-lactic-co-glycolic acid) (PLGA) nanoparticles (NPs) conjugated to the anti-EGFRvIII monoclonal antibody	EGFR receptor	DKMG/EGFRvIII cells (EGFRvIII overexpressed human glioblastoma cell line) (in vitro)	Antibody conjugated PLGA NPs were incubated with cells for 1 h and irradiated with 460 nm blue LED light at a dose of 60 J/cm^2^. Cellular uptake percentage was significantly higher in EGFRvIII overexpress cells than DK-MG^low^ cells (low expressed EGFRvIII human glioblastoma cell line).	[112]
Transferrin and aptamer conjugated [Ru(bpy)_2_(tip)]^2+^ (RBT)-loaded mesoporous ruthenium nanoparticles	Transferrin (TfR) and nucleolin expressing gliomas	U87 cells glioma cells, 293T cells and brain capillary endothelial (HBMEC) cells (in vitro), and BALB/c nude mice (in vivo)	Aptamer AS1411 and transferrin have a high binding affinity with nucleolin and transferrin receptors, respectively. Antitumor drug, RBT, has a high-efficiency PS when irradiated with 808 nm. The study suggested that the dual functionalized RBT-loaded MRN overcomes the blood-brain barrier (c), and actively targets gliomas.	[113]
Antibodies conjugated, rose bengal (RB) PS-loaded upconversion nanoparticles with a silica layer	Epithelial cell adhesion molecules (EpCAM), also known as CD326	Human colorectal adenocarcinoma HT-29 cells (in vitro)	980 nm irradiation was used to activate RB molecules. Fluorescence imaging study revealed that the synthesized antibody conjugated nanomaterials had a high affinity, with EpCAM overexpressed in HT-29 cells, and negligible in EpCAM negative murine microglia cells (BV2 cell line).	[114]

**Table 3:** Details of the photosensitizer and PDT parameters used in some of the global clinical trials of PDT in cancer treatment.

ClinicalTrials.Gov Identifier	Condition or Disease	Photosensitizer	PDT	Phase	Recruitment Status
NCT03638622	Oral cancer	Total 60 mg/kg of Aminolevulinic acid (5-ALA) administered orally via three repeated doses (20 mg/kg at 0, 1, and 2 h)	LED light sources are used in a wavelength of 405 nm. The total fluence of 100 J/cm^2^ at the lesion surface in 30–45 min	1 and 2	Completed [121]
NCT00675233	Head and neck cancer	HPPH (2-1[Hexyloxyethyl]-2-devinylpyropheophorbide-a)	665 nm	1	Completed [122]
NCT01682746	Recurrent pediatric brain tumors	Photofrin (Porfimer sodium) administered via intravenous (IV) route, 24 h before surgery and PDT	630 nm photo illumination with a total energy of 240 J/cm^2^	1	Completed [123]
NCT00984243	Lung cancer	2mm/Kg dose of Photofrin II administered via IV, 40–50 before PDT	620–630 nm, and a total energy of 200–300 J/cm^2^ (Argon-dye laser) and 100–200 J/cm^2^ (Excimer-dye laser)	NA	Completed [124]
NCT00322699	Bladder cancer	1.5 mg/kg of Photofrin (Porfimer sodium) administered via IV, 2 days before PDT	630 nm with light doses of 1200 J (±100 J)	1 and 2	Completed [125]
NCT00002975	Skin cancer	Aminolevulinic acid	633 nm laser irradiation	2	Completed [126]
NCT00862901	Breast cancer Skin cancer	Photofrin (0.8 mg/kg body weight) administered via single IV injection 36–48 h before continuous low-irradiance photodynamic therapy (CLIPT) procedure	630 nm red spectrum Diomed laser in four different experimental conditions (fluence 100, 200, 400 and 800 J/cm^2^ over 24 h) through a fiber optic patch	1	Completed [127]
NCT01800838	Lymphoma	Topically applied Silicon phthalocyanine 4	Visible light at a wavelength of 675 nm	1	Completed [128]
NCT01140178	Oral cavity carcinoma	HPPH	665 nm light with escalating laser doses from 100 J/cm^2^ to 125 and 140 J/cm^2^	1	Completed [129]
NCT02872064	Breast cancer	Single IV dose of Verteporfin (0.4 mg/kg), 60–90 min before PDT	690 nm red laser light. A light dose from 20 to 90 J/cm of light diffuser length	NA	Completed [130]
NCT01875393	Prostate cancer	Lyophilized formulation of TOOKAD^®^ (4 mg/kg)	753 nm with the fixed energy of 200 J/cm	3	Completed [131]
NCT03012009	Non-melanoma skin cancer	Methyl aminolevulinate	630 nm, 37 J/cm^2^	NA	Completed [132]
NCT02662504	Malignant Pleural Mesothelioma	Photofrin (2 mg/kg)	630 nm	2	Completed [133]
NCT01682811	Benign Dermal Neurofibromas	5-ALA	630 nm	1	Completed [134]
NCT04429139	Retinoblastoma	Verteporfin (6 mg/m^2^) administered via IV route	689 nm (200 s to generate 120 J/cm^2^)	NA	Completed [135]
NCT00868088	Squamous Cell Carcinoma	Topically applied ALA	405–420 nm at a dose of 1000 s	4	Completed [136]

## Abbreviations

µM	Micromolar
^1^O_2_	Singlet oxygen
2D	Two-dimension
3D	Three-dimension
4T1	Murine mammary carcinoma cells
4T1 cells	Mouse breast tumor cells
A375	malignant melanoma cancer cells
A549	Human adeno lung carcinoma
ALA	5-Aminolevulinic acid
Arg-Gly-Asp	Arginylglycylaspartic acid
B16-F10	Murine melanoma cells
BODIPY	boron-dipyrromethene
CACO-2 cells	Human colorectal adenocarcinoma cells
CCL1.3	Fibroblast cells
CD44	Cell surface adhesion receptor 44
CDT	Chemodynamic therapy
Ce6	Chlorin e6
CLIPT	Continuous low-irradiance photodynamic therapy procedure
DETC	Diethyldithiocarbamate
DLA cells	Dalton’s Lymphoma Ascite
DNA	Deoxyribonucleic acid
EGFRvIII	Epidermal growth factor receptor variant III
EpCAM or CD326	Epithelial cell adhesion molecules
EPR effect	Enhanced permeability and retention Effect
Fe_3_O_4_	Iron (II, III) oxide
g-C_3_N_4_ QDs	Graphitic carbon nitride quantum dots
h	Hour
H_2_O_2_	Hydrogen peroxide
HBMEC	Brain capillary endothelial cells
HBMEC	Blood-brain barrier
HEK 293T	Human embryonic kidney cells
HeLa	Human cervical carcinoma cells
HMSNs	Hollow mesoporous silica nanoparticles
HpD	Haematoporphyrin derivatives
HPPH	2-[1-hexyloxyethyl]-2-devinyl pyropheophorbide-a
HT-29 cells	Human colon cancer cells
IR820	Indocyanine green
J/cm^2^	Joules per square centimeter
Kg	Kilogram
LED	Light emitting diode
MB	Methylene blue
MCF-7	Human breast cancer cells
MDA-MB231	human breast cancer cells
MDA-MB-435	Human breast cancer cells
mg	Milligram
mTHPC	m-Tetrahydroxyphenylchlorin
MTT	2,5-diphenyl-2H-tetrazolium bromide salts
mV	Millivolt
NIH3T3 cells	Mouse embryo fibroblast cells
nm	Nanometer
NPs	Nanoparticles
O_2_	Oxygen
OEC-M1	Oral epidermoid carcinoma cells
OH	Hydroxide
PAMAM	Poly(amidoamine)
PBS	Phosphate bufferred saline
PDT	Photodynamic therapy
PEG	Poly(ethylene glycol)
PLGA	Poly(lactic-co-glycolic acid)
PPI	Poly(propylene imine)
PpIX	Protoporphyrin IX
PS	Photosensitizer
PS	Photosensitizers
PTT	Photothermal effect
RGD	Tripeptide consisting of arginine, glycine, and aspartate
ROS	Reactive oxygen species
SiHa cells	Human cervical cancer cells
SKOV-3 cells	Human adenocarcinoma cells
T98G cells	Glioblastoma cells
TAPP	5,10,15,20-tetrakis (4-aminophenyl) porphyrin
TPP	Triphenylphosphine
U87 MG	Uppsala 87 malignant glioma cells
W/cm^2^	Watt per square centimetre
ZnPc	Zinc phthalocyanine
